# The Toxic Effect of Manganese on the Acetylcholinesterase Activity in Rat Brains

**DOI:** 10.1155/2014/946372

**Published:** 2014-08-26

**Authors:** Vahid Yousefi Babadi, Leila Sadeghi, Kobra Shirani, Ali Akbar Malekirad, Mohammad Rezaei

**Affiliations:** ^1^Department of Biology, Faculty of Sciences, Payame Noor University, Iran; ^2^Department of Biochemistry, Faculty of Biological Science, Tarbiat Modares University, Tehran, Iran; ^3^Department of Toxicology, Faculty of Pharmacy, Mashhad University of Medical Sciences, Mashhad, Iran; ^4^Department of Biology, Payame Noor University, Tehran, Iran; ^5^Department of Food Safety and Hygiene, School of Public Health, Tehran University of Medical Sciences, Tehran, Iran

## Abstract

Manganese (Mn) is a naturally occurring element and an essential nutrient for humans and animals. However, exposure to high levels of Mn may cause neurotoxic effects. Accumulation of manganese damages central nervous system and causes Parkinson's disease-like syndrome called manganism. Mn neurotoxicity has been suggested to involve an imbalance between the DAergic and cholinergic systems. The pathological mechanisms associated with Mn neurotoxicity are poorly understood, but several reports have established it is mediated by changing of AChE activity that resulted in oxidative stress. Therefore we focused the effect of Mn in AChE activity in the rat's brain by MnCl_2_ injection intraperitoneally and analyzed their brains after time intervals. This study used different acute doses in short time course and different chronic doses at different exposing time to investigate which of them (exposing dose or time) is more important in Mn toxic effect. Results showed toxic effect of Mn is highly dose dependent and AChE activity in presence of chronic dose in 8 weeks reaches acute dose in only 2 days.

## 1. Introduction

Manganese (Mn) is a transition metal that is essential for normal cell growth and development [1]. Mn acts as an activator or cofactor for a variety of metalloenzymes, such as the mitochondrial superoxide-dismutase (Mn-SOD) that is a critical enzyme in suppression of oxidative stress [2], arginase, which is responsible for urea production in the liver, pyruvate carboxylase, an essential enzyme in gluconeogenesis [3], and glutamine synthetase, an astrocyte-specific enzyme that converts glutamate into glutamine [4, 5]. Additional enzymes that require Mn include hydrolases, oxidoreductases, lyases, isomerases, ligases, and transferases [6]. Clinical manifestations of Mn deficiency include seizures, retarded growth, skeletal abnormalities, and impaired reproductive function [7]. At the other end of the spectrum, excessive exposure to Mn may cause neurotoxicity [6, 8] characterized by a frequently irreversible Parkinsonian-like syndrome. Nowadays manganese has widespread application in people's life. Mn is used in the production of dyes, fertilizers, fungicides, paints, lacquers, glazes, dry batteries, fireworks, and rubber and wood preservatives. Occupational exposure to manganese occurs mainly in the mining and processing of manganese ores, alloys, and steel during welding [9]. In living system manganese is transported to organs rich in mitochondria (in particular the liver, pancreas, and pituitary) where it is rapidly concentrated [10]. But central nervous system (CNS) is one of main centers where it accumulates [11]. Manganism is linked to increased brain Mn levels primarily in brain regions known also to be iron- (Fe-) rich, namely, caudate, putamen, globuspallidus, substantianigra, and subthalamic nuclei. These regions are collectively referred to as the basal ganglia [12]. One of important effects of Mn in CNS is binding to acetylcholine esterase (AChE), modifying the substrate binding site and its inhibition [13]. AChE inhibition causes high working of cholinergic system and creates many symptoms such as increased salivation, frequent urination, vibration, and bronchial and muscle constriction. Normal activity of acetylcholinesterase (AChE) in brain is essential for brain healthy function, and changes in AChE activity are reported to be accompanied by clear signs of neurobehavioral toxicity. Therefore this parameter can be used as a neurotoxicity index in animal and human while other study can use other factors or mixed factors.

So because of more applications of Mn in industry and its dangers for nervous system in recent years, many scientists have been studying its effects in living systems that we reviewed below. The authors reported that with intraperitoneal injections of 25 MnCl_2_ mg/kg/day every 48 h, the adult rat brain acetylcholinesterase (AChE) activity is significantly changed [14]. This change in AChE activity is reported to be accompanied by clear signs of neurobehavioral toxicity (decrease in ambulation and rearings in open field as compared to controls) and significantly increased brain F4-neuroprostane levels and brain Mn-superoxide dismutase protein expression levels [14].

This study focuses on the effect of intranasal manganese administration on brain AChE activity in two acute and chronic doses and different time courses in rats. The MnCl_2_ was chosen as it is water soluble and is distributed more rapidly to the tissues than the water insoluble forms and it can be transported across synapses [15]. Manganese chloride wasa injected intraperitoneally and brain's AChE activity measured at different time intervals.

## 2. Materials and Methods

### 2.1. Animals

The animals used in this study were male Wistar rats at the age of approximately 35 days and weight of about 250–300 grams which were provided from the lab animal center of Payame Noor University, Iran. The rats are kept in special cages and have been provided with the necessary amount of food and water. Their care situations included room temperature of 23–25°C with the humidity of 55–60% and the light period was divided into 12 hours of light and 12 hours of darkness. Then they are divided in to 8 groups of 6 rats in each. Control group: rats (mean age of approximately 10 weeks) were used as a control group. In these groups the care situations were similar to the other groups with the difference that the intraperitoneal injection of manganese chloride was not done and these groups were injected by physiological serum. Acute dose was selected based on manganese concentration in the many areas of earth that human was exposed to continuously (manganese compounds and pollutions are widely distributed in air, soil, and water). Chronic dose was selected based on miner exposure or insecticide treated farming lands [16].

Three groups undergo acute dose of MnCl_2_ in 2 days; that injection was done daily (Group 1 with the dosage of 15 mg/kg, Group 2 with the dosage of 10 mg/kg, and one control group was that injected daily by 0.2 mL physiological serum).

Five groups were injected daily by chronic dose (2 mg/kg) in different time courses (Group 1: 15 days, Group 2: 30 days, Group 3: 45 days, Group 4: 60 days) and one control group for chronic dose (Group 5). MnCl_2_ injection was done intraperitoneally and daily by defined doses. After a determined time intervals rats were killed by standard method and their brains were used for AChE activity measurement.

### 2.2. Extraction of AChE from Brain Samples

24 hours after last injection rats were killed and their brains separated. Fresh brains or brains that had been frozen in liquid nitrogen and stored at −75°C were homogenized in cold buffer phosphate, 28 mM (pH 7.8) containing Triton X100 as internal standard [17]. The supernatant following centrifugation at 13,000 ×g for 20 minutes was processed; the supernatant contained AChE that was used for enzyme assay [18]. AChE (EC 3.1.1.7) activity was determined by the method of Komura [19], which is based on the formation of the yellow 5-thio-2-nitrobenzoate anion produced in the reaction between 5,5′-dithiobis-(2-nitrobenzoic acid) (DTNB) and thiocholine, after the AChE-mediated hydrolysis of ATCh. Samples were weighed (100 mg of the brain homogenate) and 1.8 mL of 28 mM buffer sodium phosphate dibasic anhydrous/sodium phosphate monobasic pH 7.8 and 0.2 mL of 5% Triton X100 were added to each tube. Briefly, the reaction was initiated after the addition of 0.05 mL of DTNB and 0.05 mL of ATCh iodide, which was used as substrate. The final concentrations of DTNB and substrate were 0.33 mM and 1.56 mM, respectively. The reaction was followed spectrophotometrically by the increase of absorbance at 430 nm. The activity was expressed as nanomole of ATCh hydrolyzed per minute per milligram protein. An aliquot of the homogenate was used for protein determination. Total protein was measured with the method of Bradford (1976). Bovine serum albumin was used as the standard. While the DTNB method has some limitations and is not fully specific, it has been widely used in assays analogous to those described herein [20]. Excitation and emission slits were both set at 5 nm. So we used absorbance changes at 430 nm for investigation of enzyme activity.

### 2.3. Statistical Analysis

For data analysis SPSS10 software was used and, in order to specify the difference between the experimental and control groups, the *t*-test was performed. Difference in the level *P* < 0.05 was considered significant.

## 3. Results and Discussion

This study for investigation of Mn effects on AChE activity used from rats that were injected by MnCl_2_ solution at different concentrations and time intervals. In first phase of study we used acute doses of MnCl_2_ (10 and 15 mg/kg) in only 2 days. As Table 1, result showed that, in presence of MnCl_2_, AChE activity showed significant increment and, in 15 mg/kg dose, enhancement is more distinguished (amount of 2918 nmol/min/mg protein for 15 mg/kg injection in comparison to 517 nmol/min/mg protein for control group).

Section 2 of this study is investigation of chronic dose of MnCl_2_ in AChE activity but in longer time intervals. Results showed when time of experiment was longer (exposure time was longer) AChE activity was more affected and more increased. This amount for 60 days experimental group was 2917 nmol/min/mg protein rather than control that is 530 nmol/min/mg protein so it increased more than 5.5 times (Table 2).

Manganese is one of the essential elements for animals and human but exposure of them to high concentration of Mn causes accumulation of this element in CNS that occurs in miners, welders, ferroalloy workers, battery manufacturers, and car mechanics. Manganism, the poisoning effect of Mn, is neurological brain disorder that involved CNS. Previous studies investigated the effects of short-term exposure to Mn on the adult rat brain antioxidant status and the activities of AChE, noting that AChE activity was significantly increased in the Mn exposed group [14]. Herein, we have used an* in vivo* model to test the effect of Mn different dose in AChE activity in rat brains. Our results showed that, after 8-week exposure to chronic Mn dose (2 mg/kg), AChE activity increased more than 5.5 times, that is obtained by acute dose exposuse (15 mg/kg) in only 2 days. The neurotoxic events of Mn are mediated by central cholinergic systems [21]. It is hypothesized that the Parkinson's-like movement disorder inherent to excessive exposure to Mn results from imbalance between the DAergic and cholinergic systems of the basal ganglia [14]. The delicate functional neurochemical balance is disrupted upon damage to both dopamine (DA) and cholinergic systems, caused either directly or indirectly via GABAergic and glutamatergic interneurons.* In vivo* studies showed that Mn alters the activity of enzymes involved in cholinergic transmission, such as AChE and choline acetyltransferase [22]. AChE is the degradative enzyme of ACh and thereby is responsible for the termination of cholinergic response in muscarinic and nicotinic brain ACh receptors [23]. Therefore studies demonstrated that AChE activity is modulated by Mn exposure by increasing or decreasing and imbalanced between the DAergic and cholinergic systems [14]. Laboratory animal (rat) responses to Mn exposure are variable; some studies reported that AChE activity was significantly reduced in the striatum of rats treated with MnCl_2_ [24, 25], and some cases are in agreement with present study and showed increased AChE activity by exposure to excessive Mn concentration [26]. The probable reasons for this variation are several factors, such as age, dose, route of administration, genetic background, and frequency of exposure as well as the time between sacrifice and the freezing of the brain or the inactivation of the enzymes [14]. But it is important that all of the studies are in agreement with imbalance created in presence of Mn. It is interesting that human exposure to excessive Mn always decreased AChE activity. As discussed above most of previous studies on rat showed that: (1) Mn stimulates AChE concomitant with significant changes in the levels of biomarkers of oxidative stress, (2) AChE is a target of Mn in the central nervous system that may trigger or contribute to the development of oxidative stress, (3) the stimulatory effect of Mn on AChE activity promotes increased levels of neuronal oxidative stress and neuroinflammatory biomarkers, and (4) AChE activity may serve as an early biomarker of Mn neurotoxicity [14], and our results confirmed those statements. Exposure to excessive levels of Mn may cause a variety of neurotoxic effects that involve changing in biomarkers of oxidative stress as a result of changing in AChE activity [27].

## 4. Conclusion 

It is important that exposure of human to excessive Mn creates decreased activity of AChE so it disrupted the balance between DAergic and cholinergic systems. Different results between experiments on human and rat were possibly made by structural alteration or different genetic background and mentioned reasons between these two enzymes. Therefore, the utility of AChE activity can be used as an early biomarker of Mn neurotoxicity; it should be further addressed in biomonitoring Mn neurotoxicity. Also results showed that enhancement of this biomarker is dose dependent.

## Figures and Tables

**Table 1:** AChE activity differences in presence of acute dose of Mn in short time (2 days). Asterisk symbols present significant changes (*P* < 0.01).

Rat groups	AChE activity (nmol/min/mg protein)
Control	517 ± 89
Acute dose (15 mg/kg)	2932 ± 385*
Acute dose (10 mg/kg)	2787 ± 205*

**Table 2:** AChE activity variations in presence of chronic dose (2 mg/kg) of Mn in the different long time intervals. Asterisk symbols indicate significant changes (*P* < 0.01).

Rat groups	AChE activity (nmol/min/mg protein)
Control	5270 ± 41
Chronic dose in 15 days	567 ± 49
Chronic dose in 30 days	1976 ± 212*
Chronic dose in 45 days	2179 ± 238*
Chronic dose in in 60 days	2923 ± 251*
